# Quality assessment of shoulder MRI according to practice parameters of American College of Radiology: A multi-center study in Jordan

**DOI:** 10.25122/jml-2022-0351

**Published:** 2023-03

**Authors:** Mohammad Ayasrah, Izzeddin Qtaish

**Affiliations:** 1 Department of Allied Medical Sciences, Jordan University of Science and Technology, Ramtha, Jordan; 2 Radiology and Interventional Radiology Department, Faculty of Medicine, Jordan University of Science and Technology, Ramtha, Jordan

**Keywords:** MRI-shoulder, radiology audit, quality control, shoulder pathology, ionization, ACR, ACR – American College of Radiology, ESSR – European Society of Musculoskeletal Radiology, FOVf – Field of view along the frequency encoding direction, FOVp – Field of view along the phase encoding direction, MRI – Magnetic Resonance Imaging, NC-MRI – Non-contrast magnetic resonance imaging, STIR – Short Tau Inversion Recovery

## Abstract

Magnetic resonance imaging (MRI) is essential for assessing shoulder conditions. This study aimed to evaluate current shoulder MRI practices in Jordan, including technical parameter patterns, and determine if they adhere to the American College of Radiology (ACR) guidelines. The retrospective analysis included data from 48 eligible participants from 13 MRI centers in March 2021. Descriptive and correlation data analysis were performed using IBM SPSS statistics version_20 and Excel 2013. Most MRI centers (50%) were private outpatient clinics with closed MRI machines above 1 Tesla. Most participants (62.5%) were male, and shoulder pain (47.9%) was the main clinical indication. Most shoulder orientations (68.7%, 33/48) were right shoulders, and the coronal MRI planes (43%, 121/280) were the most common. The alignment percentage for the axial plane was 100%, but MRI artifacts of the shoulder were present in 8.2% of cases (23/280). Dark fluid T1-W coronal sequence was not conducted in 25% of the cases. The percentage of the field view (FOV) within ACR recommendations was 45% (126/281), and slice thickness parameters were 96% (269/281). The recommended pixel area for all sequences was 47.9% (134/280), encompassing all axial, sagittal oblique, and coronal planes. However, crucial parameters, such as FOV and slice thickness, were inadequate and did not meet the ACR guidelines, resulting in suboptimal image quality of shoulder MRI. To improve MRI image quality, it is recommended that MRI technologists receive ongoing education and training on appropriate MRI image parameters.

## INTRODUCTION

The shoulder is a complex joint system characterized by the greatest range of motion and the least stability in the musculoskeletal system [1]. As the optimal imaging modality varies for the shoulder, imaging techniques play an important role in diagnosing and treating various pathologies and injuries [2]. Non-contrast magnetic resonance imaging (NC-MRI) is the leading and most accurate imaging modality for evaluating, detecting, assessing, and staging shoulder pathology, allowing excellent visualization of the soft tissues forming the shoulder that are often the source of shoulder pathology [3,4]. The success of shoulder MRI as a golden modality for diagnosing MRI pathology depends on the technical quality of this procedure [5,6]. The American College of Radiology (ACR) has developed evidence-based criteria and technical standards guidelines for shoulder MRI practice to evaluate the quality and appropriateness of shoulder MRI imaging [7]. These recommendations are intended to reduce shoulder MRI overutilization, which has been reported to range from 27% [8] to as high as 45% in some studies [9]. The American College of Radiology (ACR) emphasizes the importance of properly performing and interpreting MRI, which not only aids in the diagnosis but also treatment planning and prognostication [7]. However, it should be performed only for valid medical reasons and after careful consideration of alternative diagnostic modalities [10]. ACR has established adequacy factors for successful accreditation of shoulder MRI [7]. These factors are categorized into three levels. The first level category includes plane coverage, slice alignment, and orientation (Table 1). Failure to meet these recommendations can result in the rejection of accreditation.

**Table 1:** ACR recommendations for anatomy coverage and slice orientation for the basic shoulder MRI plane.

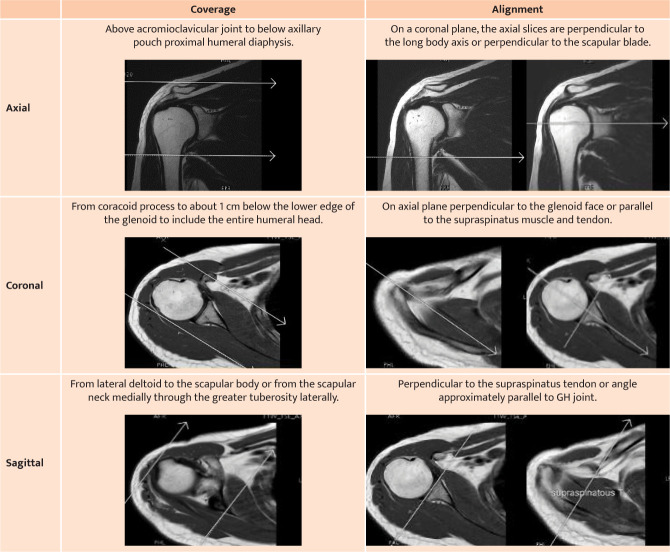

The level 2 category focuses on the sequences protocol that controls the signal intensity and image contrast of the tissues. As clinical requirements continue to evolve, imaging sequences are constantly being modified to address more specific clinical needs [11]. On average, MRI shoulder protocols consist of 4-5 sequences [12]. The ACR requires at least four mandatory sequences. These include two coronal oblique planes, one fluid-sensitive sequence with a suppressed fat signal (T2W FS), and another dark fluid sequence (T1W). The other two sequences are a proton density for axial (Long TR, Short TE PD) and a bright fluid for sagittal (T2W) [13].

The particular imaging parameters (i.e., TR, TE, FA, ETL, etc.) and the type of pulse sequence are not specified and are left to the preference of the imaging facility [14]. Nonetheless, fast 2D spin-echo sequences are currently the choice for evaluating shoulder joint during non-contrast MRI [1]. Currently, the T1-weighted sequence can be acquired as a fast spin-echo acquisition with optimized scanning parameters to depict sharp anatomy definition and reduce blurring. The fluid-sensitive sequence with the suppressed fat signal can be obtained with proton density fast spin echo or T2* gradient echo or inversion recovery method to stop fat movement, such as STIR (short tau inversion recovery) [2]. The outcome signal intensity from this sequence is the bright signal for fluid and a darker signal than fluid for fat.

The third level category is concerned with spatial resolution. Table 2 shows the main parameters recommended by ACR.

**Table 2:** Recommend parameters of spatial resolution for shoulder MRI.

Parameter	ACR recommended value
FOV (max)	≤16 cm
Number of phase encoding steps (Np)	162
Number of frequency encoding steps (Nf)	256
Slice thickness (ST)	≤4 mm
Inter-slice gap	0.8 mm
In-plane pixel (read)	≤0.7 mm
In-plane pixel (phase)	≤1.0 mm
Pixel area	≤0.8 mm^2^

ACR guidelines recommend a maximum field of view (FOV) of 16 cm or less and a maximum slice thickness of 4mm or less, with a slice gap not exceeding 20%. For higher magnetic strength, a slice thickness of 3.5 mm or less is recommended for the oblique sagittal and oblique coronal planes to achieve high spatial resolution and better visualization of fine details such as tendon, labrum, and articular cartilage pathology. The literature review shows very limited articles about the adequacy factors of shoulder MRI. A retrospective study [15] on the adequacy of MRI of the shoulder in tertiary care was conducted and was limited to only two main parameters of the ACR factors, adequacy coverage and adequacy planes. The study showed 100% coverage for all axial, oblique coronal, and oblique sagittal planes. The adequacy of the slice alignment was 63% and 84% for oblique sagittal and oblique coronal, respectively. Another retrospective study was conducted to assess the quality of the arm position during shoulder MRI [16]. The study showed that one-third of shoulder MRIs were done in a supine position with the hand in internal rotation, which reduced the quality of the procedure for better diagnoses. However, many articles discussed the appropriateness criteria for shoulder MRI [17-19]. This study aimed to thoroughly examine the current practices of non-contrast shoulder MRI against the well-established ACR's recommended criteria to identify any discrepancies between actual and standard practices. The objective of this study was to evaluate current shoulder MRI practices in Jordan, assess technical parameter patterns, and determine if they meet the standards set by the ACR. A retrospective analysis was conducted on demographic data, clinical history, and the adequacy of shoulder MRI to investigate this.

## Material and Methods

### Data collection

This study was conducted in multiple hospitals and private radiology clinics. An invitation was sent to 85 radiology departments to participate, where they were informed of the study's objectives and guaranteed anonymity. They were asked to submit 4 cases of shoulder MRIs on anonymized CDs performed in March 2021. The retrospective data was gathered from 48 eligible cases of shoulder MRIs performed at 13 MRI centers out of 52 initially recorded cases after eliminating those with damaged CDs or prior shoulder surgery.

### Analysis parameters

To evaluate the adequacy of shoulder MRI, this study used the guidelines provided by the ACR. The guidelines categorize the evaluation into three levels, and Table 1 lists the parameters for the anatomical coverage and plan alignment categories. Meeting these criteria is essential to proceed with the evaluation of other types. The second level of evaluation pertains to the adequacy of the MRI sequence. The ACR recommends that at least four MRI sequences should be performed in three orthogonal planes. A case is considered failed if there is a loss of any planes or sequences.

Table 2 displays five technical parameters used to measure spatial resolution. The pixel area should not exceed 0.8 mm^2^, which can be calculated by the formula ((FOVp/Np)*(FOVf/Nf)), where ((FOVp/Np)) represents the in-plane pixel (phase) of 1.0 mm, and (FOVf/Nf) represents the in-plane pixel (read) of 0.7 mm.

### Equipment

To facilitate the review and analysis of MRI cases, a special cloud account was used to upload the CD cases to The Netherlands Stratus B.V. PACS system. RadiAnt. Viewer 2020.2 DICOM viewer software was also used to examine the technical information of the MRI image (FOV, slice thickness and spacing, matrix size, phase, and frequency encoding) through the DICOM tag option. IBM SPSS statistics version 20 and Excel 2013 were used for data tabulation, descriptive analysis, and correlation.

## Results

Out of the 85 MRI centers invited to participate in the study, only 13 MRI centers in Jordan agreed to take part, resulting in a total of 48 shoulder MRI cases. The response rate was 15.3%. A sample size of 13 centers yielded a margin of error of +25% (at the 95% confidence interval).

### Demographic characteristics

Most MRI centers (50%) were private outpatient clinics, and 16.7% (8/48) reported operating compact MRI machines below 1 Tesla. 62.5% (30/48) of the cases were male, with a mean age of 44.9 years (SD=14.6, range 21-80 ). The main clinical indications for the shoulder MRI were pain (47.9%, 23/48), limitation of motion (20.9%), and rotator cuff tear (16.7%), and the remainder were post-operative tendinopathy and trauma. Most shoulder orientation (68.7%, 33/48) was the right shoulder.

### Coverage and alignment

The axial plane, oblique sagittal plane, and oblique coronal plane imaging were used to assess the coverage of the shoulder MRI according to the standards in Table 1. All of these sequences were evaluated and found to be 100 percent adequate in terms of coverage.

**Figure 1:**
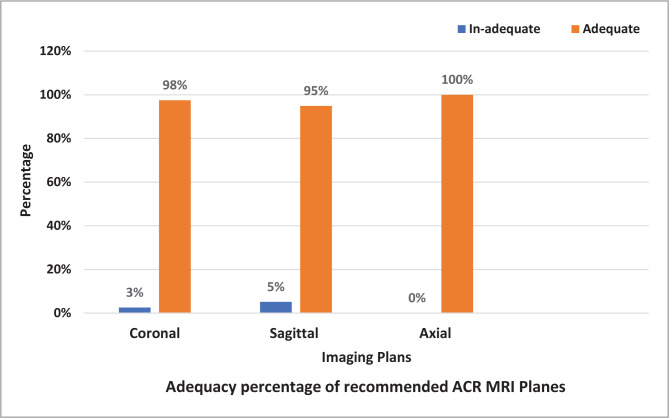
Adequacy percentage of recommended ACR MRI planes.

Alignment adequacy was assessed for the coronal, sagittal, and axial planes using ACR standards in Table 1, with a total number of 280 MRI sequences conducted across 48 cases. The majority of sequences (43%,121/280) were coronal MRI planes. The percentage of alignment adequacy was 100% for the axial plane, followed by 97.5% for the coronal and 94.9% for the sagittal plane, as depicted in Figure 1.

### ACR recommended sequences and planes

**Figure 2:**
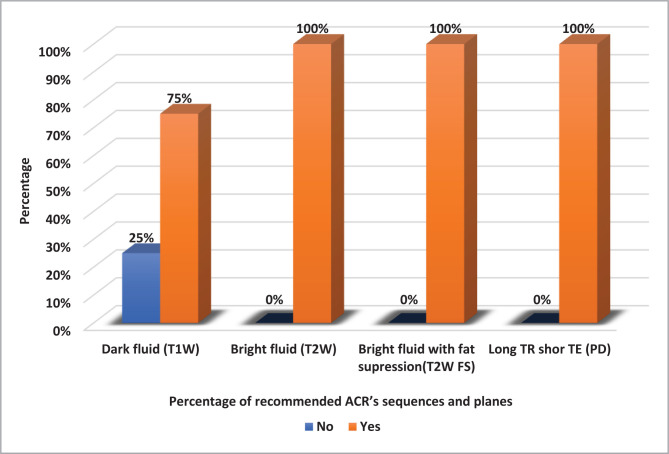
ACR-recommended sequences and planes.

On average, each patient underwent 5.8 MRI sequences with a total of 280 sequences conducted at a rate of 43% (121/280) on a coronal oblique plane, while the rest were distributed almost equally (28%) for sagittal oblique and axial planes. ACR recommends at least four MRI sequences for procedure adequacy, as shown in Table 2. The study showed that 25% of the cases failed to conduct dark fluid T1W coronal sequence, as illustrated in Figure 2.

### Spatial resolution

**Figure 3:**
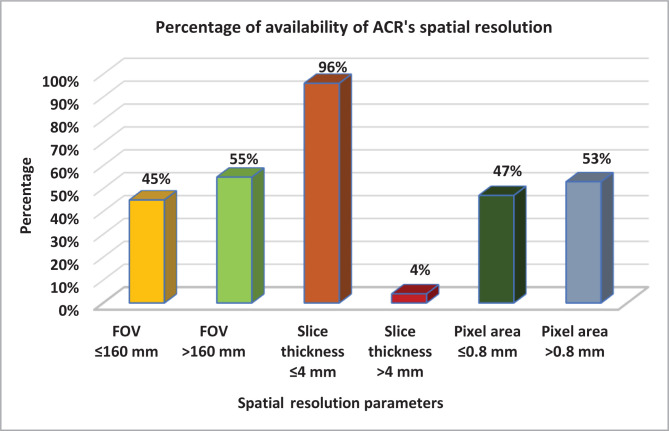
ACR-recommended spatial resolution availability.

The methodology section outlines the steps taken to quantify the MRI spatial resolution and slice thickness, which were assessed and compared to ACR’s recommendations. The percentage of the FOV (≤ 16 mm) within the ACR’s recommendation was 45% (126/281), whereas the slice thickness parameters (≤ 4 mm) were 96% (269/281). The recommended pixel area (≤0.8mm) was observed in 47.9% (134/280) of all sequences, which encompassed all axial, sagittal oblique, and coronal planes, as depicted in Figure 3.

The distribution of FOV accepted parameters, the slice thickness, and the pixel area according to imaging planes were also assessed. The accepted FOV values were very similar across all planes, and the highest was for the sagittal plane. The slice thickness of 4 mm or lesser was the highest for the sagittal plan (100%). The recommended pixel area was about 48% for all planes, shown in Table 3.

**Table 3:** Adequacy percentage of FOV, slice thickness, and pixel area per ACR’s recommendation.

Plane	FOV ≤160 mm		Slice thickness ≤4 mm		Pixel area ≤8 mm^2^	
	Count	%	Count	%	Count	%
Axial	32	50%	77	95%	37	48%
COR	56	55%	117	94%	60	48%
Sag	37	63%	75	100%	36	48%
Total	125	45%	269	96%	133	48%

The cross-tabulation of FOV, the slice thickness, and the pixel area with the MRI environment were also assessed. Table 4 demonstrates that none of the shoulder MRIs conducted on open MRIs (0.0%) met the ACR's recommended FOV criteria.

**Table 4:** Adequacy percentage of FOV, slice thickness, and pixel area per MRI environment.

MRI Type	FOV ≤160mm		Slice Thickness ≤4 mm		Pixel area ≤8 mm^2^	
	Count	%	Count	%	Count	%
Closed	125	45%	225	80%	133	48%
Open	0	0%	44	16%	0	0%
	125	45%	269	96%	133	48%

Although 45% of the MRI sequences were performed on closed MRI, they still did not meet recommended parameters as per ACR's guidelines. Of 269 adequate MRI sequences, 225 (80%) had a slice thickness of 4mm or less. Only 48% of the MRI sequences matched the recommended pixel area (≤ 8 mm^2^), and these were conducted on closed MRI machines with 1 Tesla or greater. However, all the MRI sequences conducted on open MRI machines were inadequate and failed to meet the recommendations.

## Discussion

The preferred noninvasive method for assessing shoulder joint injuries is a combination of clinical examination and MRI [20]. MRI is an excellent tool for evaluating bony, cartilaginous, ligamentous, and synovial diseases, as well as trauma, infection, and malignancy [21,22]. Improving the accuracy of diagnosing shoulder abnormalities is highly dependent on the quality of MRI images. MRI image quality is determined by a range of factors, including the Signal to Noise Ratio (SNR) and Contrast to Noise Ratio (CNR), as well as adherence to the practice parameters set by relevant radiology societies [23].

To properly evaluate the shoulder joint, MRI imaging must be performed in multiple planes. The American College of Radiology (ACR) guidelines for routine shoulder MRI studies require at least four sequences with different contrast weightings taken in three imaging planes, including axial, oblique coronal, and oblique sagittal, to enable better evaluation and diagnosis of shoulder pathologies [24]. Each imaging plane has its advantages when examining different anatomic components. Generally, MRI shoulder examination starts with the axial plane using proton density sequences with fat suppression to obtain transverse images for evaluating the subscapularis tendon, glenohumeral joint, glenoid labrum, and bicipital groove contents [25]. Dark fluid (T1-weighted) and bright fluid with fat suppression oblique (T2 or PD weighted) images in coronal oblique planes as well as bright fluid (T2-weighted) images in sagittal oblique are required by ACR to evaluate the labrum, biceps tendon, AC joint, rotator interval, supraspinatus, and infraspinatus muscles/tendons.

In this study, MRIs of the shoulder were obtained on all three planes for each patient, with an average of 6 sequences acquired in the axial, oblique sagittal, and oblique coronal planes. The scanning time was approximately 20 minutes, which is within the maximum limit of 40 minutes set by ACR standards. In comparison, a study by Subhas et al. found that the mean standard protocol time for shoulder MRI was 4 minutes and 33 seconds for 3T MRI and 15 minutes and 40 seconds for 1.5T MRI [26].

The study showed that all sequences (100%) conducted in axial and sagittal oblique were performed according to ACR guidelines. Only 25% of dark fluid (T1-weighted) sequences performed in coronal planes failed to match the ACR’s guidelines. On the other hand, all bright fluid with fat suppression (T2 or PD weighted) sequences in oblique coronal planes fully matched the standards of the ACR. Several studies from different countries other than the USA encourage using the same ACR practice parameters [24,25,27,28].

All the shoulder joint structures must be fully covered to reduce the likelihood of missing any abnormalities [7]. On all sequences and planes of MRI, shoulder anatomy was considered 100% covered. This was in line with the findings in Pakistan, which also showed 100% accuracy for all plans and sequences [15] as recommended by the Royal College of Radiologists [29] and the American College of Radiology [30]. Researchers at Warrington and Halton Teaching Hospitals NHS Foundation Trust found that coronal oblique coverage was 100% accurate, while axial and sagittal oblique coverage were 69% and 72% accurate, respectively [30].

Slice orientation and direction are extremely important for depicting anatomy and preventing structure superimposition. In most MRI sequences and planes, slice orientation is determined by the supraspinatus muscle and tendon, especially in coronal and sagittal oblique planes, as shown in Table 1. According to the findings of this study, all axial planes had 100% slice orientation, while coronal planes had a 97.5% slice orientation rate, and sagittal planes had a 94.9% slice orientation, consistent with the findings of Altaf [15]. The accuracy of sagittal oblique orientation was 91%, and 94% for coronal oblique orientation at Warrington and Halton Teaching Hospitals NHS Foundation Trust. The adequacy of the slice orientation and direction for all studies was lesser than the 100% target of ACR [30].

Optimizing spatial and contrast resolution can be achieved by controlling different technical parameters, including slice thickness, inter-slice gap, image matrix, and FOV. ACR has published several editions of clinical image quality guidelines over the past two decades to define and quantify MRI spatial resolution using different determinants and formulas, as explained in the methodology section [13]. The FOV is inherently related to spatial determination, which is the key parameter that determines the quality of MRI images. For this reason, FOV should be kept at 16 cm or less. However, the FOV parameter in this study was inadequate in most cases (55%, 155/280), specifically in axial and coronal planes conducted in open MRI. Another important factor affecting image quality is slice thickness. The study found that in almost all cases (96%), a slice thickness of 4 mm or less was used. Matrix size is also a crucial parameter determined by the frequency and phase encoding. The pixels area of the image matrix measured in mm^2^ summarizes the frequency encoding, phase encoding, and the FOV in one figure and should be equal to or less than 0.8 mm^2^, as shown in the methodology section. All the sequences (53%, 148/280) conducted in open MRI were inadequate, with a value above 0.8 mm^2^. Open MRI with a magnetic field of less than 1 Tesla has low SNR to produce high-resolution shoulder images. ACR recommends using larger FOVs and smaller matrix sizes on account of spatial resolution that may limit the sensitivity of the shoulder MRI examination.

The present study has some limitations that should be acknowledged. The results may not be generalizable to other countries or regions due to variations in MRI practices. Therefore, the findings of this study may be more useful as an internal quality control initiative within the government.

## Conclusion

MRI of the shoulder is a useful noninvasive diagnostic tool that provides detailed images of various structures within the shoulder joint. Accurate diagnosis relies heavily on obtaining high-quality images determined by several technical parameters, including anatomical coverage, slice orientation, and more. However, this study found that important parameters such as slice thickness and FOV were below the recommended values by ACR guidelines, resulting in suboptimal image quality. Thus, it is recommended that MRI technologists receive continuous education on proper MRI image parameters. ACR guidelines should be disseminated to radiographers and radiologists, and radiographers should be educated on the anatomy of the rotator cuff, as well as the importance of aligning the localizer parallel to the supraspinatus central tendon to ensure visualization of the rotator cuff tendons in continuity.

## Personal thanks

We express our gratitude to our research assistants for their valuable contributions to collecting the data.

## References

[1] Lee SB, Kim KJ, O’Driscoll SW, Morrey BF, An KN (2000). Dynamic glenohumeral stability provided by the rotator cuff muscles in the mid-range and end-range of motion. A study in cadavera. J Bone Joint Surg Am.

[2] Zoga AC, Kamel SI, Hynes JP, Kavanagh EC (2021). The Evolving Roles of MRI and Ultrasound in First-Line Imaging of Rotator Cuff Injuries. AJR Am J Roentgenol.

[3] Saragaglia D, Barthomeuf C, Banihachemi JJ (2021). Deciphering acute shoulder trauma with normal initial X-ray: Contributions of ultrasonography and MRI. Orthop Traumatol Surg Res.

[4] Karami A, Ghezelbash P, Qorbanisani M, Ghezelbash Z (2020). Non-contrast MRI Findings of Adhesive Capsulitis: A review. Rheumatology Research.

[5] Shin YK, Ryu KN, Park JS, Jin W (2018). Predictive Factors of Retear in Patients With Repaired Rotator Cuff Tear on Shoulder MRI. Am J Roentgenol.

[6] Henninger HB, Christensen GV, Taylor CE, Kawakami J (2020). The Muscle Cross-sectional Area on MRI of the Shoulder Can Predict Muscle Volume: An MRI Study in Cadavers. Clin Orthop Relat Res.

[7] American College of Radiology (2021). ACR–SPR–SSR Practice parameter for the performance and interpretation of magnetic resonance imaging (MRI) of the shoulder. Published online.

[8] Al hadidi F, Ryalat N, Alryalat SA, Hadidy A (2019). Shoulder Magnetic Resonance Imaging Part 1: Descriptive Frequency and Outcome in a Teaching Hospital. Jordan Med J.

[9] Sheehan SE, Coburn JA, Singh H, Vanness DJ (2016). Reducing Unnecessary Shoulder MRI Examinations Within a Capitated Health Care System: A Potential Role for Shoulder Ultrasound. J Am Coll Radiol.

[10] Galvez-Sánchez CM, de la Coba P, Duschek S, Reyes Del Paso GA (2020). Reliability, Factor Structure and Predictive Validity of the Widespread Pain Index and Symptom Severity Scales of the 2010 American College of Rheumatology Criteria of Fibromyalgia. J Clin Med.

[11] Yuan J, Poon DMC, Lo G, Wong OL (2022). A narrative review of MRI acquisition for MR-guided-radiotherapy in prostate cancer. Quant Imaging Med Surg.

[12] Nigues A, Salentiny Y, Nabergoj M, Lädermann A, Neyton L (2022). The Digitation Sign Facilitates Diagnosis of Shoulder Subscapularis Lesions on Preoperative Magnetic Resonance Imaging. Arthroscopy, Sports Med and Rehabil.

[13] American College of Radiology (2021). MRI Exam-Specific Parameters: MSK Module (Revised 10-182021). Published online October 18.

[14] He X, Tan C, Tan V, Li K Recursive 3D Segmentation of Shoulder Joint with Coarse-scanned MR Image. arXiv preprint.

[15] Altaf MO, Shafqat M, Gul P, Siddique K (2020). Audit on adequacy of magnetic resonance of the shoulder in a tertiary care oncological setup Pakistan. PJR.

[16] Davis DL, Faddoul DG, Almardawi R (2017). Practice Quality Improvement for Patient Positioning on Shoulder MRI to Reduce Potential Diagnostic Errors. J Am Coll Radiol.

[17] Bouëtté A, Karoussou-Schreiner A, Ducou Le Pointe H, Grieten M (2019). National audit on the appropriateness of CT and MRI examinations in Luxembourg. Insights into imaging.

[18] Amini B, Beckmann NM, Beaman FD, Wessell DE (2018). ACR Appropriateness Criteria® Shoulder Pain–Traumatic. J Am College Radiol.

[19] Reynolds AW, Armstrong AD (2017). Appropriateness and Suggested Use of MRI in Management of Shoulder Pain. J Musculoskelet Disord Treat.

[20] Jäschke M, Köhler HC, Weber MA, Tischer T (2021). Subacromial impingement syndrome: association of multiple magnetic resonance imaging parameters with shoulder function and pain. Arch Orthop Trauma Surg.

[21] Arora S (2021). Imaging Modalities for Rotator Cuff and Labro-Ligamentous Complex of Shoulder Joint Evalution. Annals of RSCB.

[22] Gottsegen CJ, Merkle AN, Bencardino JT, Gyftopoulos S (2017). Advanced MRI techniques of the shoulder joint: current applications in clinical practice. AJR Am J Roentgenol.

[23] Mulyati S, Rusyadi L, Rahmawati IMP, Wibowo AS, Indrati R (2019). Differentiation Image Quality of MRI Shoulder Joint with Variation RF Coils. E3S Web of Conferences.

[24] Aydingoz U, Canbulat N, Demirhan M (2014). Radiological Assessment of the Shoulder Region. Turk J Phys Med Rehab.

[25] Ashir A, Lombardi A, Jerban S, Ma Y (2020). Magnetic resonance imaging of the shoulder. Pol J Radiol.

[26] Subhas N, Benedick A, Obuchowski NA, Polster JM (2017). Comparison of a Fast 5-Minute Shoulder MRI Protocol With a Standard Shoulder MRI Protocol: A Multiinstitutional Multireader Study. AJR.

[27] Bellelli A, Silvestri E, Barile A, Albano D (2019). Position paper on magnetic resonance imaging protocols in the musculoskeletal system (excluding the spine) by the Italian College of Musculoskeletal Radiology. Radiol Med.

[28] Lee HS, Lee YH, Jung I, Song OK (2020). Optimization of MRI protocol for the musculoskeletal system. Journal of the Korean Society of Radiology.

[29] Doweidar A, Murphy A, Elsakaan M, Hashmi M (2022). Audit of adequacy of the large joints magnetic resonance imaging. Clinic Radiol RCR.

[30] Dutt S, Ganguly A Assessing the adequacy of magnetic resonance imaging of the shoulder. Warrington and Halton NHS Foundation Trust.

